# Gut Microbiome-Based Diagnostic Model to Predict Diabetes Mellitus

**DOI:** 10.1080/21655979.2021.2009752

**Published:** 2021-12-19

**Authors:** Hai-Tao Yang, Jing-Kun Liu, Wen-Juan Xiu, Ting-Ting Tian, Yi Yang, Xian-Geng Hou, Xiang Xie

**Affiliations:** a Department of Cardiology, First Affiliated Hospital of Xinjiang Medical University, Urumqi, China; b Department of Oncology, First Affiliated Hospital of Xinjiang Medical University, Urumqi, China; c College of Basic Medical Science, Xinjiang Medical University, Urumqi, China

**Keywords:** Intestinal microorganisms, diabetes, diagnostic model, fasting blood glucose, 16S rRNA genes

## Abstract

The aim of this study was to determine the diversity of intestinal microflora and its correlation with clinical parameters in diabetic patients and healthy subjects and to assess the importance of intestinal flora in patients with diabetes. Forty-four patients with diabetes were included. The control group included 47 healthy people. Their data, biochemical indicators and results from 16S rRNA sequencing of their fecal samples were collected. Compared with the healthy population, the intestinal flora of the diabetic patients was obviously abnormal. Within the diabetes group, the abundances of the genera Faecalibacterium, Prevotella, and Roseburia were higher, and the abundances of the genera Shigella and Bifidobacterium were lower. In the correlation analysis between bacteria and clinical indicators, it was found that the genera Veillonella and unclassified_Enterobacteriaceae were negatively related to blood glucose, while the genera Phascolarctobacterium, unidentified_Bacteroidales and Prevotella were significantly positively correlated with fasting blood glucose. Twelve microbial markers were detected in the random forest model, and the area under the curve (AUC) was 84.1%. This index was greater than the diagnostic effect of fasting blood glucose. This was also supported by the joint diagnostic model of microorganisms and clinical indicators. In addition, the intestinal flora significantly improved the diagnosis of diabetes. In conclusion, it can be concluded from these results that intestinal flora is essential for the occurrence and development of diabetes, which seems to be as important as blood glucose itself.

**Abbreviations:** PCoA: principal coordinate analysis; NMDS: non econometric multidimensional scaling analysis; LEfSe: linear discriminant analysis effect size; LDA: linear discriminant analysis; POD: probability of disease; BMI: body mass index; DCA: decision curve analysis

## 1. Introduction

According to a report from the World Health Organization, diabetes mellitus has become an epidemic in the 21^st^ century and is the third most serious chronic lifelong disease that threatens human health after tumors and cardiovascular diseases. The World Health Organization predicts that the number of people with type 2 diabetes will double to at least 350 million worldwide by 2030 unless appropriate action is taken [1]. The complications of diabetes mellitus, especially the chronic complications of the disease, can involve multiple organs and lead to disability and a high mortality rate, seriously affecting the physical and mental health of patients and imposing a heavy burden on individuals, families and society. Although the diagnosis and treatment methods of diabetes mellitus have been constantly updated in recent years, this has not yet prevented the increasing trend of the diabetes incidence rate [2]. Therefore, it is crucial to further explore the new mechanisms.

For many years, research on intestinal microorganisms has been unlimited to digestive tract diseases such as *Clostridium difficile* infection. Further research has revealed that the relationship between the host and intestinal microorganisms is not a simple parasitic relationship but rather a mutually beneficial symbiotic relationship [3]. As early as the early twentieth century, some researchers hypothesized that there are approximately 100 trillion bacteria in the intestinal flora of a normal person, the total number of which is equivalent to the total number of human cells. The enormous genome contained in these microorganisms has been called the human ‘Second Genome’ [4,5]. Subsequent studies have continuously confirmed that this large genome is closely related to the host’s birth, aging, illnesses and death. It has been reported that the host’s long-term diet [6], smoking [7], drinking [8] and even stress [9,10] and obesity [11] will change the structure of the intestinal flora, and changes in the structure of the flora will further lead to changes in its microbial metabolites. These specific compounds can affect and promote the occurrence and development of diseases. For instance, trimethylamine N-oxide is closely related to cardiovascular diseases, diabetes and other chronic diseases [12].

The relationship between diabetes and intestinal microflora is receiving increasing attention. There are also reports on the structure of the intestinal flora in diabetic patients. For example, in a recent clinical study of 180 people, researchers compared the intestinal microorganisms of a diabetic group and healthy people. At the genus level, the relative abundances of Prevotella and Alloprevotella were significantly higher in the diabetes group [13]. In a prospective study in the same year, 341 samples were assessed. Lower abundances of *Bifidobacterium longum, Coprobacillus unclassified*, and *Veillonella dispar* and higher abundances of *Roseburia hominis, Porphyromonas bennonis*, and *Paraprevotella unclassified* were found in diabetic patients [14]. Although many studies have been reported, most studies have reported only the intestinal flora associated with diabetes and did not clarify the importance of intestinal microbes in the occurrence and development of diabetes. Fasting blood glucose plays a crucial role in the occurrence and development of diabetes. The aim of this study was to build a diabetes diagnosis model based on the important intestinal microflora found in diabetic patients and healthy people. This model was used to study the importance of intestinal flora in the occurrence and development of diabetes through comparison with fasting blood glucose, which strongly proved that the intestinal flora is closely related to the occurrence and development of diabetes. It also provides a new effective target for the diagnosis and treatment of diabetes.

## 2. Methods

### 2.1. Research design and patient population

Ninety-one samples were included, among which 44 were from diabetic patients and 47 were from healthy people. Among these, the diagnosis of diabetes mellitus was based on the guidelines for the prevention and treatment of type 2 diabetes in China (2020 edition). The diagnosis of hypertension in the population was based on the *Hypertension Guidelines* in China in 2020, and the number of people diagnosed with hypertension was 67. The 91 patients included all underwent coronary angiography. Moreover, coronary heart disease was diagnosed in patients when at least one main coronary artery showed stenosis of greater than 50%, which revealed a total of 72 cases of coronary heart disease.

The clinical data of the patients were collected, including the basic information and biochemical indices of the enrolled population. Exclusions of patients were as follows: 1. patients diagnosed with heart failure, structural heart disease, and pulmonary heart disease; 2. patients with a history of using antibiotics or probiotics within 3 months; 3. patients with severe liver and kidney dysfunction, such as patients whose creatinine levels were not less than 2-fold of the normal upper limit and patients whose aspartate transaminase or alanine transaminase levels were not less than 3-fold of the normal upper limit; 4. patients with an abnormal stool morphology, such as diarrhea and dry stools. The study design complied with the Declaration of Helsinki and was approved by the Ethics Committee of the First Affiliated Hospital of Xinjiang Medical University. Before recruitment, informed consent was obtained from eligible patients.

### 2.2. Past history and clinical data

For alcohol intake, subjects who drank more than eight standard drinks per week were classified as heavy drinkers [3]. For smoking, the World Health Organization’s definition was adopted. Those who had smoked continuously or cumulatively for 6 months or more were defined as smokers (current smoking status). Smokers who no longer smoked at the time of the survey and had a cessation of smoking for more than 6 months were given a past smoking status [3]. Five milliliters of peripheral venous blood was taken from each patient after 12 hours of fasting. The laboratory testing data included routine blood testing parameters, blood biochemical analysis results, renal function and liver function parameters, and blood lipid analysis results.

### 2.3. Fecal specimen collection, DNA extraction, and sequencing

A disposable sterile bag was provided for each participant to collect feces, and a fecal sampler with a spoon (X517, Xinkang, Jiang Su, China) was also provided for sample collection. All participants were trained to collect the samples and instructed to drain their urine before retaining the feces and to fully wash hands and wear disposable gloves before taking the fecal sample. Fecal samples newly collected from each participant were divided into five equal parts of 200 mg, immediately transported to the laboratory and frozen at −80°C [15]. The bead-beating method was utilized to isolate the bacterial DNA from fecal samples as described previously [16].A polymerase chain reaction was used to amplify the V3-V4 region of the 16S rRNA genes by using the extracted DNA from each sample as the template. The DNA extraction and sequencing were performed by Shanghai Personal Biotechnology Co., Ltd. (http://www.personalbio.cn, Shanghai, China). The sequencing data were processed using the Quantitative Insights Into Microbial Ecology (v1.8.0) pipeline, as described previously [17].The data that support the findings of this study have been deposited in the NCBI (https://submit.ncbi.nlm.nih.gov/) with accession number SRP346048.

### 2.4. Microbiome data analysis

When exploring the microbial diversity of both groups, dimension reduction technology in bioinformatics was used [3]. For the analysis of alpha diversity, assessing the richness and diversity of the sample was evaluated by the Chao1, Shannon, observed-species and Faith’s PD indices. The Chao1 and observed-species indices were utilized for the community’s abundance [18]. The Shannon index was applied to assess the community’s diversity, and Faith’s PD index was calculated in combination with the diversity of the flora to assess the kinship between the species of the floras [19,20]. For the analysis of beta diversity, the Bray–Curtis distance and unweighted UniFrac distance were used for calculations [3]. In the commonly applied microbial beta diversity calculation distance, the Bray–Curtis distance pays more attention to the change in microbial abundance, while the unweighted UniFrac distance pays more attention to the evolutionary relationship of the microorganisms [21,22]. Beta diversity was demonstrated by principal coordinate analysis (PCoA) and noneconometric multidimensional scaling analysis (NMDS). For the statistical tests between the two groups, adonis was used [3]. For determining which species were different, linear discriminant analysis effect size (LEfSe) analysis was used, which is a nonparametric Kruskal–Wallis and Wilcoxon rank sum test combined with linear discriminant analysis (LDA) [23]. For the correlation between bacterial abundance and biochemical indicators, the Spearman correlation coefficient was used. In addition, for the screening of marker bacteria, a mechanical learning model was used to screen important bacteria [3]. For the calculation of the probability of disease (POD) index, first, the logarithmic transformation of bacterial abundance was carried out, and then the probability was calculated in combination with logistic regression as the pod index [24].

### 2.5. Statistical analysis

SPSS version 22 (SPSS Inc., Chicago, IL, USA) and R version 3.2.4 (R Foundation for Statistical Computing, Vienna, Austria) were used for the statistical analysis of clinical data. For LEfSe analysis, the Galaxy online analysis platform was used [3]. Continuous variables were analyzed by the Student’s t test. The chi-square test or Fisher’s exact test was used to analyze categorical variables. All results were considered statistically significant when p values were less than 0.05.

## 3. Results

To explore the correlation between intestinal flora and diabetes, 16S rRNA gene sequencing was used to analyze the intestinal flora of diabetic patients and healthy people. There were significant differences in the composition of intestinal flora between diabetic patients and healthy people. There was a significant correlation between specific intestinal flora and fasting blood glucose. The importance of these different bacteria was ranked. It was found that the top 12 intestinal flora had a good classification efficiency in diagnosing disease, even exceeding the diagnostic efficiency of fasting blood glucose. Through the combined analysis of intestinal microbial markers and clinical variables, we found that the effect of combined clinical indicators was better. These results indicate that the intestinal microflora plays an important role in the development of diabetes in the course of disease development.

### 3.1. Basic population information and intestinal microbial composition

A total of 91 patients were enrolled, of whom 44 were diagnosed with type 2 diabetes. For the control group, the patients were matched with the diabetic group based on age, sex, body mass index (BMI), living habits (smoking, drinking) and potential diseases, and 47 patients were ultimately included. The baseline characteristics of the entire patient group are shown in Table 1. The average age of the enrolled patients ranged from 58 years old to 59 years old. The average BMI in the two groups ranged from 24 to 26. The two smoking populations accounted for 38.64% and 34.04% of the entire patient group, and the drinking populations accounted for 27.27% and 27.66% of the entire patient group, respectively. In the included experimental group and the control group, the coronary heart disease population accounted for 79% and 78% of the total population in each group, respectively, and the hypertensive population accounted for 70% and 76% of the total population in each group, respectively. Since the basic information of the population matched in the long-term oral drug status records and biochemical examination index statistics of the patients, there were no significant differences that were found, except for the differences in the treatment with diabetes drugs and the fasting blood glucose.

**Table 1.** Baseline characteristics of study population

Variable	2 Diabetes(n = 44)	Control(n = 47)	P value
Age, yr	58 ± 8	59 ± 12	0.556
Male	24(54.55%)	28(59.57%)	0.393
Body mass index, kg/m^2^	26.06 ± 3.639	24.55 ± 3.159	0.61
Smoking	17(38.64%)	16(34.04%)	0.406
Drinking	12(27.27%)	13(27.66%)	0.577
Basic diseases			
Coronary heart disease	35(79.55%)	37(78.72%)	0.565
Essential hypertension	31(70.45%)	36(76.60%)	0.335
Long term oral/subcutaneous injection medication			
Metformin	40(90.9%)	/	/
Glucosidase inhibitors	11(25%)	/	/
DDP-4 inhibitors	2(4.55%)	/	/
Insulin	16(36.36%)	/	/
Aspirin	33(75%)	34(72.34%)	0.481
Clopidogrel	30(68.18%)	25(53.19%)	0.106
Stain	32(72.72%)	26(55.32%)	0.065
β-block	27(61.36%)	28(59.57%)	0.516
Calcium Channel Blockers	12(27.27%)	7(14.89%)	0.116
ACEI or ARB^a^	18(40.91%)	18(38.30%)	0.484
Laboratory results			
White blood cells, ×10^9^/L	6.76 ± 1.9	6.67 ± 1.98	0.838
Neutrophilicgranulocyte,%	59.91 ± 11.36	57.49 ± 13.39	0.354
Hemoglobin, ×10^12^/L	136.50 ± 21.34	138.55 ± 13.47	0.587
Platelets, ×10^9^/L	235.34 ± 65.98	221.85 ± 53.13	0.284
blood urea nitrogen,mmol/L	5.37 ± 1.91	5.05 ± 1.32	0.366
Creatinine,umol/L	62.00(46.34–72.00)	64.00(44.20–72.50)	0.948
Uric acid,umol/L	314.45 ± 91.63	320.28 ± 77.47	0.746
FBG^b^,mmol/L	6.59(5.77–9.43)	4.7(4.27–5.395)	<0.001
Total cholesterol, mmol/L	3.64 ± 0.90	3.69 ± 1.13	0.849
Triglyceride, mmol/L	1.72(1.11–2.39)	1.38(1.01–2.02)	0.184
LDL^c^,mmol/L	2.20 ± 0.77	2.20 ± 0.92	0.972
HDL^d^,mmol/L	1.01 ± 0.27	1.15 ± 0.27	0.13
Total bilirubin,umol/L	10.1(7.4–14.4)	10.6(8.3–14.8)	0.553

Data are presented as median (interquartile range), mean ± SD, or number (%).

ACEI or ARB^a^: angiotensin converting enzyme ingibitors or angiotensin receptor blocker.

FBG^b^: fasting blood glucose; LDL^c^: low density lipoprotein; HDL^d^: high density lipoprotein.

**Figure 1.**
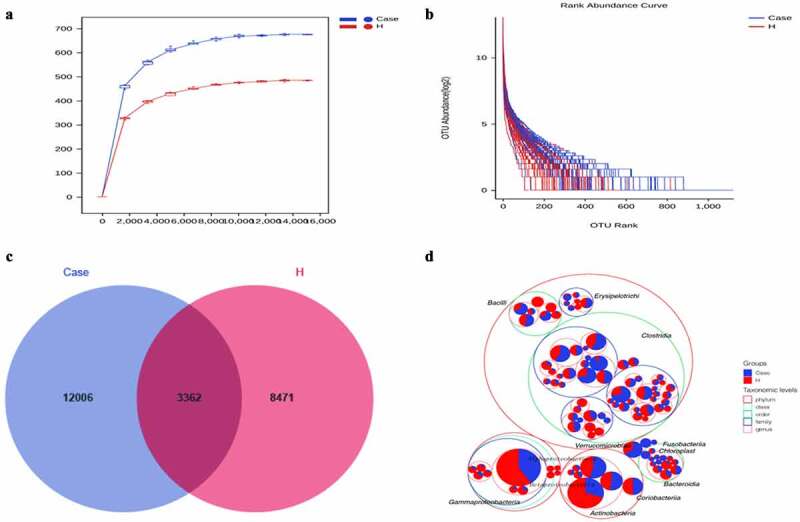
Increased fecal microbial diversity in patients with DM vs healthy controls.(a)Rarefaction Curve.(b) Rank abundance curve.(c) Venn diagram displaying the overlaps between groups showing that 3362 of the total richness of 23,839 OTUs were shared between the CAD patients and the healthy controls. OTUs: operational taxonomy units.(d) Circle packing chart

**Figure 2.**
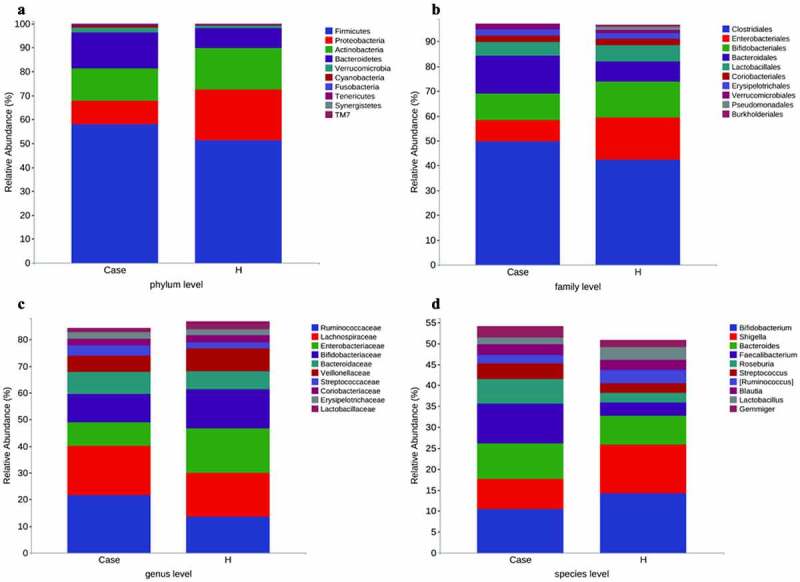
Stacked histogram of species composition in the top 10 of each group. (a) Phylum level. (b) Family level. (c) Genus level. (d) Species level

The stool collected from the included population was analyzed for intestinal microorganisms. 16S rRNA sequencing was performed in 91 patients. To determine whether the sample size was sufficient and to estimate the abundance of the community, a species dilution curve and an abundance grade curve were used (Figure 1(a, b)). From these curves, it can be seen that they all appeared stable, indicating that the included sample size was sufficient for statistical analysis. Through sequencing data analysis, we identified approximately 20,000 OTUs from the discovery cohort. Venn diagrams displaying the identified OTUs are shown in Figure 1(c). The results indicated that the OTU level in the diabetes group was significantly higher than that in the control group. Species annotation was carried out for OTU levels in the top 100 most abundant taxa and for Clostridia, Gammaproteobacteria, Actinobacteria, and Bacteroidia (Figure 1(d)). Then, the top 10 bacterial abundances of species at the phylum level, family level, genus level and species level were annotated. The comparison in the histogram revealed that the species composition between the two groups was quite different (Figure 2(a‒d)). Among the bacteria at the phylum level, the specific top three bacterial proportions in the experimental group were Firmicutes (57.91%), Bacteroidetes (15.08%), and Actinobacteria (13.3%), while the specific top three bacterial proportions in the control group were Firmicutes (51.32%), Proteobacteria (21.09%), and Actinobacteria (17.38%).

### 3.2. A specific gut microbial signature in type 2 diabetes mellitus patients

**Figure 3.**
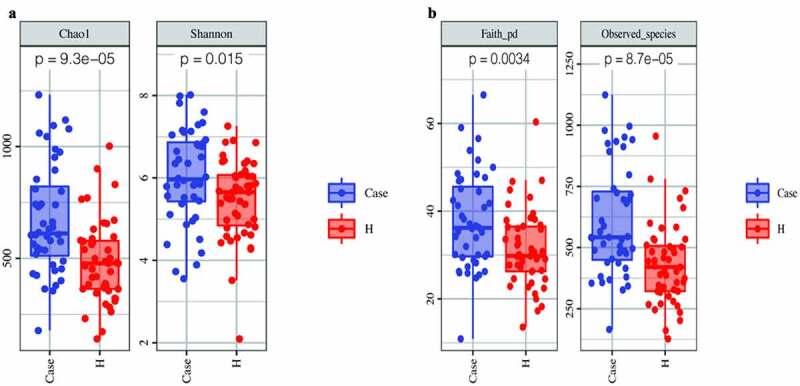
Comparison of fecal microbial diversity, as estimated by the Chao1 index and Shannon index (a), Faith-pd index and Observed_species index (b)

**Figure 4.**
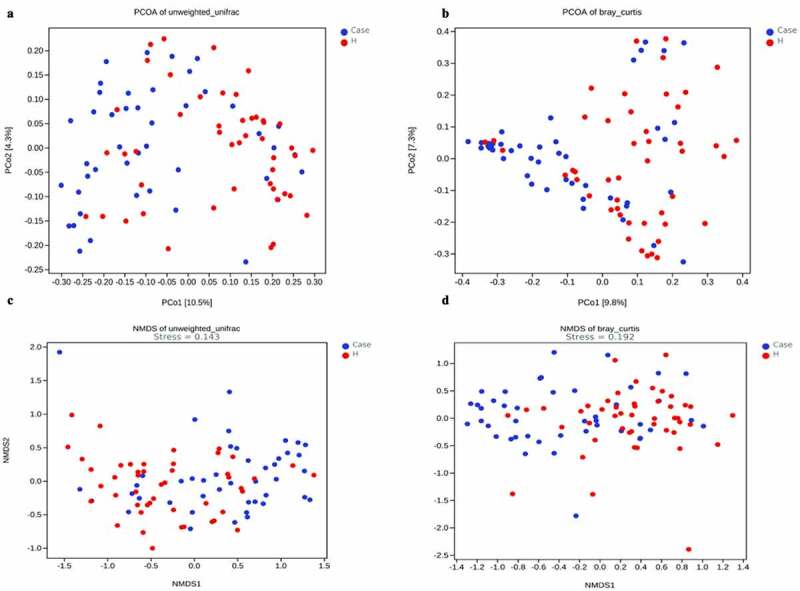
Beta diversity was calculated using unweighted UniFrac [left] or brau_curtis [right] by PCoA and NMDS, indicating a symmetrical distribution of fecal microbial community among all the samples

**Figure 5.**
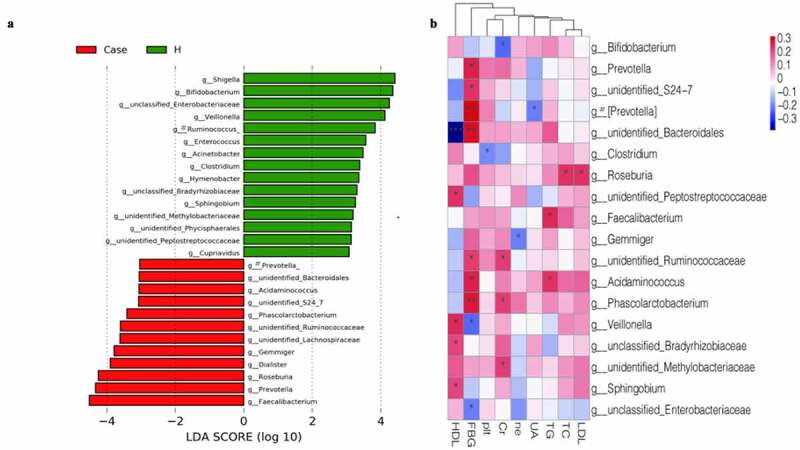
Phylogenetic profifiles and differences of gut microbes between patients and healthy controls. [A] LEfSe method identifified the most differentially abundant taxons between the patients and healthy controls. [B] Heatmap of correlation between differential bacteria and clinical indicators. #: Tentative names in greenenes database

The alpha diversity analysis of the two groups is presented (Figure 3(a‒b)). The results indicated that there were significant differences between the diabetes group and the control group. Furthermore, the Chao1, observed-species and Shannon indices were high in the diabetes group, suggesting a greater abundance of bacterial communities in the diabetes group than in the control group. The Faith’s PD index was higher in the diabetic group, suggesting a greater affinity between bacteria in the diabetic group. Beta diversity analysis between the two groups was based on unweighted UniFrac distance and Bray–Curtis distance. The beta diversity analysis results are presented in the form of PCoA and NMDS dimension reduction (Figure 4(a‒d)). In the NMDS analysis, stress equaled 0.143 (< 0.2) on the basis of the unweighted UniFrac distance and 0.192 (<0.2) on the basis of the Bray–Curtis distance. In addition, adonis analysis showed that R^2^ = 0.031 and P = 0.001. All the species diversity analyses indicated significant differences between the two groups. Moreover, in the diabetes group, the species richness was higher than that in the control group, and the genetic distance between bacteria was larger than that in the control group. These results suggested that during the development of diabetes, the composition of the gut microbiota might change. LEfSe analysis was performed for different species between the two groups where the LDA threshold was greater than 3. The results showed that there were significant differences in 27 species of bacteria at the genus level between the two groups. In the diabetes group, the abundances of the genera Faecalibacterium, Prevotella and Roseburia were very high, while the abundances of the genera Shigella and Bifidobacterium were very low (Figure 5(a)). Subsequently, the correlation between these different bacteria and clinical biochemical indicators was analyzed and displayed in the form of heatmaps. Except for the genera Veillonella and unclassified_Enterobacteriaceae, which were negatively correlated with blood sugar, the genera Phascolarctobacterium, unidentified_Bacteroidales, and Prevotella had an obvious positive correlation with fasting blood glucose. Among these indicators, in addition to the greater correlation with blood sugar that was observed, a correlation with high-density lipoprotein was also obvious. For instance, the abundance of the genus unidentified_Bacteroidales had a significant negative correlation with high-density lipoprotein (Figure 5(b)).

### 3.3. Identification of microbial genus-based markers of 2DM

**Figure 6.**
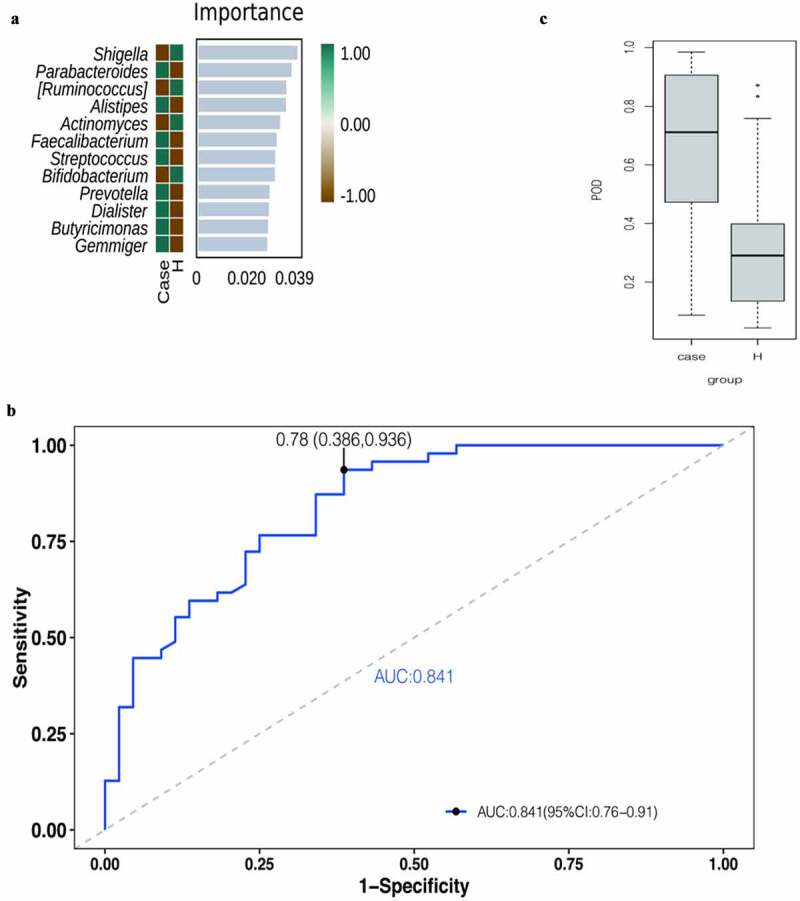
Important biomarkers. [A] The top 12 bacteria belong to the genus level. [B] ROCs curve with AUC for the diagnostic performance of the gut microbial model. [C] Comparison of the POD of gut microbiome. [Ruminococcus] Temporary name

To identify important markers for diabetes, a 10x cross-examination (10-fold cross-validations) of genus-level bacteria was performed to find further important signature species through random forests in mechanical learning models. Twelve bacteria of high importance were found at the genus level, including Shigella and Parabacteroides, as shown in Figure 6(a). Subsequently, the 12 bacteria were used to diagnose diabetes and draw the ROC curve (Figure 6(b)). The cutoff value of the ROC curve was 0.78, the 95% confidence curve was 0.386–0.936, and the AUC area was 0.841. The probability of disease (POD) index was calculated by applying the identified optimal set of 12 genera. As shown in Figure 6(c), the biomarker could correctly predict 84.1% of the diagnoses between the diabetes group and the control group, indicating that this indicator has a good diagnostic effect on the disease.

To evaluate the diagnostic significance of intestinal flora to diabetes, the clinical indices in the included population were screened for diagnostic markers. As shown in Table 2, the only clinical indicators with a p value less than 0.05 and an area under the ROC curve (AUC) greater than 0.5 were hypertension and fasting blood glucose. There were more people diagnosed with diabetes among people with high blood pressure; specifically, the AUC area of the diagnosis of hypertension for diabetes was 0.658 (p < 0.019, 95% Cl: 0.533–0.783). The diagnostic curve and AUC area of a patient’s random fasting blood glucose for diabetes was 0.839 (p < 0.001, 95% Cl: To evaluate the diagnostic significance of intestinal flora to diabetes, the clinical indices in the included population were screened for diagnostic markers. As shown in Table 2, the only clinical indicators with a p value less than 0.05 and an area under the ROC curve (AUC) greater than 0.5 were hypertension and fasting blood glucose. less than that of the intestinal flora (0.839 < 0.841).

**Table 2.** Candidate variables for clinical model development

Variables	AUC	P values	95% CI
FBG	0.839	0.000	0.742–0.937
HDL	0.337	0.016	0.212–0.462
EH	0.658	0.019	0.533–0.783
BMI	0.613	0.095	0.481–0.744
CAD	0.599	0.140	0.471–0.728
Ne	0.589	0.189	0.458–0.720
SMOKING	0.552	0.437	0.421–0.684
LDL	0.451	0.469	0.318–0.584
TG	0.546	0.496	0.414–0.678
TC	0.457	0.527	0.326–0.589
WBC	0.463	0.580	0.330–0.595
Cr	0.471	0.663	0.336–0.605
UA	0.474	0.698	0.340–0.608
SEX	0.475	0.706	0.342–0.607
TBIL	0.477	0.737	0.343–0.612
AGE	0.480	0.766	0.345–0.614
PLT	0.519	0.774	0.383–0.656
HBL	0.491	0.898	0.356–0.627
DRINGKING	0.503	0.962	0.371–0.635

WBC: white blood cells; Cr: creatinine; UA: uric acid; PLT: platelet count; HBL: haemoglobin level;

FBG: fasting blood glucose; HDL: high density lipoprotein; BMI: body mass index; TG: triglyceride;

TC: total cholesterol; TBIL: total bilirubin; LDL: low density lipoprotein; Ne: neutrophil percentage.

CAD: coronary artery disease; EH: essential hypertension.

The included index was 44/47, and there was no missing value.

### 3.4. Nomogram prediction of DM

**Figure 7.**
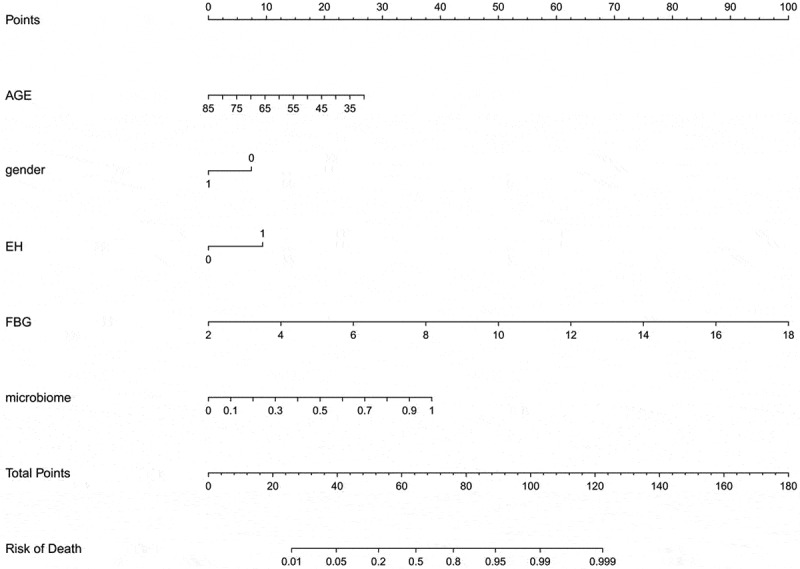
Instructions for using the nomogram

**Figure 8.**
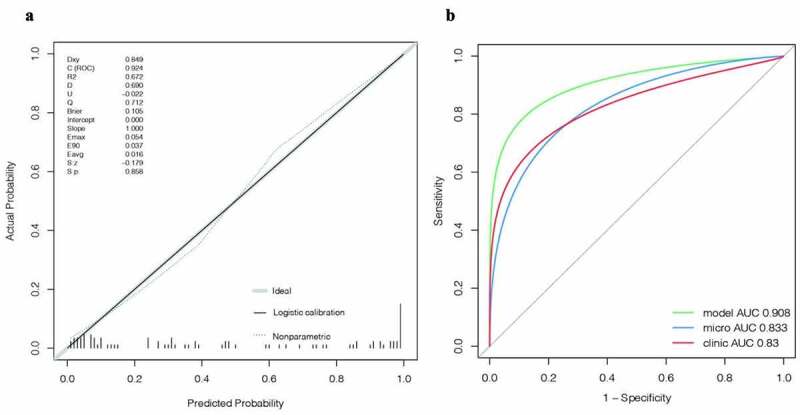
[A] Calibration curve. [B] The AUCs for the diagnostic performances of the clinical model, microbiome model and the combined model

To optimize the diagnostic efficiency, the microbial abundance at the 12 species level was transformed logarithmically, and the POD value was calculated in combination with logistic regression [24]. A nomogram diagnostic model was constructed in combination with age, sex (if female), and clinical variable indicators such as denial from the diagnosis of hypertension and fasting blood glucose (Figure 7). A patient was randomly selected from the population. This patient was a 45-year-old woman who had been diagnosed with high blood pressure. Her biochemical index fasting blood glucose was 6 mmol/L. The stool was tested for landmark microorganisms, and the POD value was calculated to be 0.7. Combining the above information into this diagnostic model, the probability of diabetes in the patient was 95%. The results are shown in Fig. S1. To verify the effectiveness of the diagnostic model, a C-index curve was drawn. As shown in Figure 8(a), the C-index of this model was 0.924, which indicates an excellent effect on the diagnosis of diabetes. The ROC curve was drawn according to the clinical index, microbial pod index and the combined model. The effectiveness of the POD index for the independent diagnosis of diabetes was also greater than that of the clinical index model including fasting blood glucose. Interestingly, the combined AUC area of the two reached 0.908, which suggests that microbial indicators can optimize clinical indicators for disease diagnosis (Figure 8(b)). In addition, the decision curve analysis (DCA) curve was drawn, and similar conclusions were drawn from the DCA curve (Fig. S2).

## 4. Discussion

In this study, sample patients were included after strict screening. Clinical indices and feces were collected from the included patients, and the feces were sequenced by 16S rRNA. Through in-depth analysis of intestinal flora and clinical indicators, it was found that there was a significant difference between the intestinal flora of diabetic patients and the healthy population and that the diagnosis of diabetes based on intestinal flora was even better than that based on fasting blood glucose. For the first time, a diagnostic model of intestinal microbiota combined with clinical indicators was established in diabetic patients. When the diagnostic model was verified, the C-index was close to 1. This shows that the diagnostic effect of this model is very good. Through these results, we can infer that the intestinal flora is very important for the occurrence and development of diabetes, and its importance seems to be as great as that of blood sugar itself. This result has also been supported in previous studies. For instance, Awgichew et al. attributed differences between diabetes and healthy groups to the role of short-chain fatty acid-producing bacteria [25]. Zhang et al. have reported on patients in the diabetes group at the genus level of bacteria. They found that the relative abundance of Prevotella and Alloprevotella was significantly higher [13]. This is consistent with the reports of significant differences in intestinal flora between diabetic and healthy people in this study. The specific differences in bacterial levels are not consistent with recent studies, and it is considered that most patients have underlying diseases. Actually, in the common process of clinical diagnosis and treatment, similar patients are more common, which is more in line with the complexity of patients in the process of clinical practice and more suitable for clinical diagnosis and treatment.

In this study, the abnormal abundance of the genus Parabacteroides (the intestinal microorganism) in diabetic patients seemed to have a significant effect on the diagnosis of diabetes. This is not the first time that the relationship of the genus Parabacteroides with diabetes has been reported. In a recent prospective randomized controlled study in Spain, the relationship between diet, gut microbes and diabetes was explored. In the study results, the role of the genus Parabacteroides in affecting the occurrence and development of diabetes was confirmed. This effect seems to be related to the enhancement of metabolic pathways, including terpenoid-quinone, lipopolysaccharide and N-glycan biosynthesis [26]. The genus Alistipes was also shown to be very important in the diabetic group, and the effect of the genus Alistipes on diabetes has also been reported recently [27]. The genus Alistipes is currently considered to be a bacterium that produces short-chain fatty acids (SCFAs), which have a number of potential roles in modulating metabolic health and DM risk factors, such as blood glucose regulation and metabolic regulation, and maintaining the integrity of the intestinal barrier [25]. There were also effects on the genus Faecalibacterium in diabetic patients, and the different bacterial distributions were more obvious in the diabetic group. Currently, more studies have been reported on the genus Faecalibacterium, and the abnormal abundance of the genus Faecalibacterium was also reported by different institutes in experimental groups [28,29]. In the present study, the abundance of the genus Faecalibacterium was positively associated with the presence of triglycerides, which was presumed to be likely related to the immune effects of the genus Faecalibacterium. A similar finding has also been mentioned in previous studies [30]. In this study, the genus Streptococcus was also selected as important in randomized forest models in the diabetes group. The genus Streptococcus is widely found in nature, in human and animal stools, and in the nasopharynx and intestines of healthy people and can mainly cause suppurative inflammation, toxin diseases and hypersensitivity-reactive diseases. This has also been reported in diabetes in previous studies [27]. In the present study, there were two Prevotella genera in bacterial abundance and found to be clinical indicators, which was the result of the current Greenggenes database. Both Prevotella genera were annotated, which also emphasizes the relationship between Prevotella and blood glucose. In the present study, the abundance of the genus Prevotella was high in the diabetes group and was positively associated with the clinical biochemical index of fasting blood glucose. This conclusion was also mentioned in a 2020 study in the United States. In this study, the researchers attributed this effect to lipopolysaccharide, which is a component of the gram-negative bacterial wall. It can activate the local immune response and may cause low-grade systemic inflammation, leading to insulin resistance and affecting the occurrence and development of diabetes [31]. In addition to the genera mentioned above, the genera Dialister, Butyricimonas and Gemmiger were shown in the random forest model of the diabetes group. This conclusion has been confirmed in different studies [32,33]. In the heatmap of differential bacteria and biochemical indicators, it was suggested that the genus Gemmiger was surely negatively correlated with the percentage of neutrophils, suggesting that the genus Gemmiger is likely to play a role through immune regulation.

Among the 12 types of bacteria included in the model, the genera Shigella, Ruminococcus, Actinomyces, and Bifidobacterium were relatively important in the control group. This is not the first time that Shigella has been included in diabetes-related diagnostic models. In a recent study on diabetic nephropathy, the study model included 25 genera of bacteria for diagnosing diabetic nephropathy, and the AUC area of the diagnostic model after drawing the ROC curve was 0.972. In such a model, the genus Shigella was also emphasized. However, the mechanism of action of the genus Shigella in diabetes or diabetes-related diseases is currently unclear. The genus Ruminococcus is closely related to the diet structure of the host. This bacterium has also been reported in diabetes. This conclusion is similar to the outcome of this study [33]. The genus Actinomyces is widely distributed in nature and has a wide variety of species. It is a member of the normal flora of the human body and can cause endogenous infections. There are few reports of the genus Actinomyces in the diagnosis model of diabetes. In 2019, a Chinese study reported the microbial structure of patients with hyperlipidemia and gestational diabetes. The report revealed that the abundance of this genus was abnormal between the two groups, which also verifies these results [34]. Among these genera with high abundance in the control, the genus Bifidobacterium should be the most reported microbial bacteria, as it has been clearly defined as a probiotic. It has been added to dairy products for consumption and plays a role in the pharmaceutical industry. In the LEfSe analysis and research results, the genus Bifidobacterium had the highest LDA in the control group. This conclusion does not seem to be a surprise. The improvement in blood sugar due to Bifidobacterium was also mentioned in a study. Elderly patients with type 2 diabetes took 200 ml of a compound drink containing *Bifidobacterium bifidum* every day. After taking it for 1 month, the fasting blood glucose level was significantly reduced [35]. However, at present, there are few reports on the mechanism by which Bifidobacterium improves blood sugar, which should be the future research direction of probiotics for improving disease. In addition, there are also research reports on the negative correlation between the genus Bifidobacterium and creatinine. In a randomized controlled study in Brazil in 2020, the subjects were given drugs containing the Bifidobacterium genus regularly. After follow-up, it was found that the blood creatinine level was different between the two groups and that the creatinine level of the experimental group taking the drug decreased significantly [36].

The present study has several limitations that may require close attention. First, although an accurate diagnostic model has been developed by using intestinal flora, the function of the microbiota is not clear. This study did not assess bacterial metabolites, and the research on the mechanism is insufficient. Second, the included sample size was small and was not verified in different regions and different time periods. Third, the test method was 16S gene sequencing. In the database, there are still many tentative names and unidentified and unclassified bacteria. It is suggested that multiple group methods, such as macrogenes, be further explored.

## 5. Conclusion

Intestinal flora affects diabetes. Among the diabetic population, the abundance of Faecalibacterium, Prevotella and Roseburia was relatively high, while the abundance of Shigella and Bifidobacterium was low. Veillonella and unclassified_ Enterobacteriaceae were negatively correlated with blood glucose, and Phascolactobacterium, unidentified_ Bacteroidales and Prevotella were positively correlated with fasting blood glucose. In addition, the intestinal flora distinguished between diabetes and healthy people well, and this ability to distinguish diabetes was as good as that of fasting blood glucose as an indicator. Intestinal flora combined with clinical indicators was shown to be more effective in diagnosis.

## Supplementary Material

Supplemental Material

## Highlights

- The intestinal flora of diabetic patients is significantly different from that of healthy people.
- The abundance of intestinal bacteria in diabetic patients is higher than that in healthy people.
- The influence of intestinal flora in diabetic patients is as great as that of fasting blood glucose.
