# Supplementary material for: Gut Microbiome-Based Diagnostic Model to Predict Diabetes Mellitus
Source: Bioengineered. 2021 Dec 19;12(2):12521–34. doi: 10.1080/21655979.2021.2009752 (PMC8810174; doi:10.1080/21655979.2021.2009752)
Supplement: Supplemental Material [file KBIE_A_2009752_SM7534.zip › supplementary/Supplemental figure.docx]

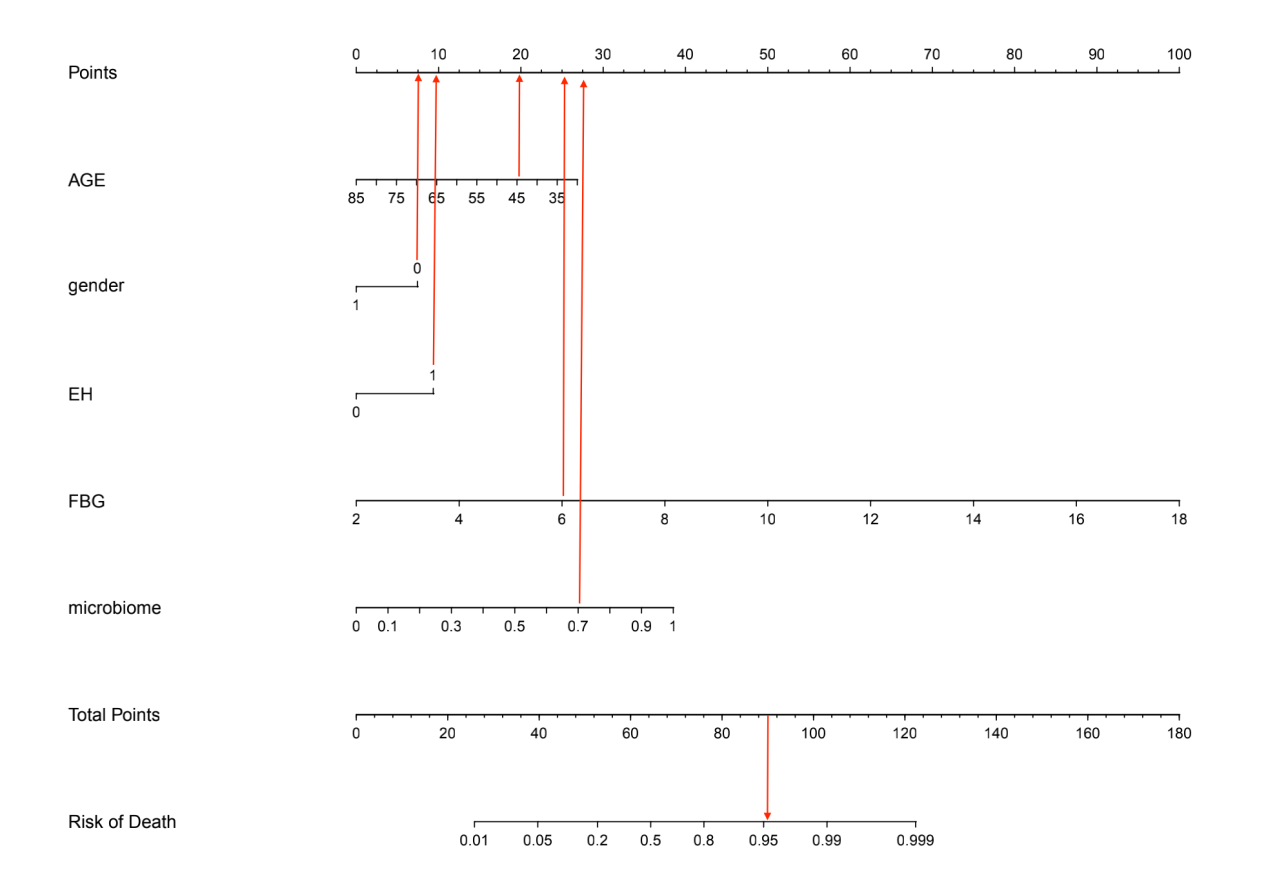


Supplemental figure 1. Use the nomogram specifically.


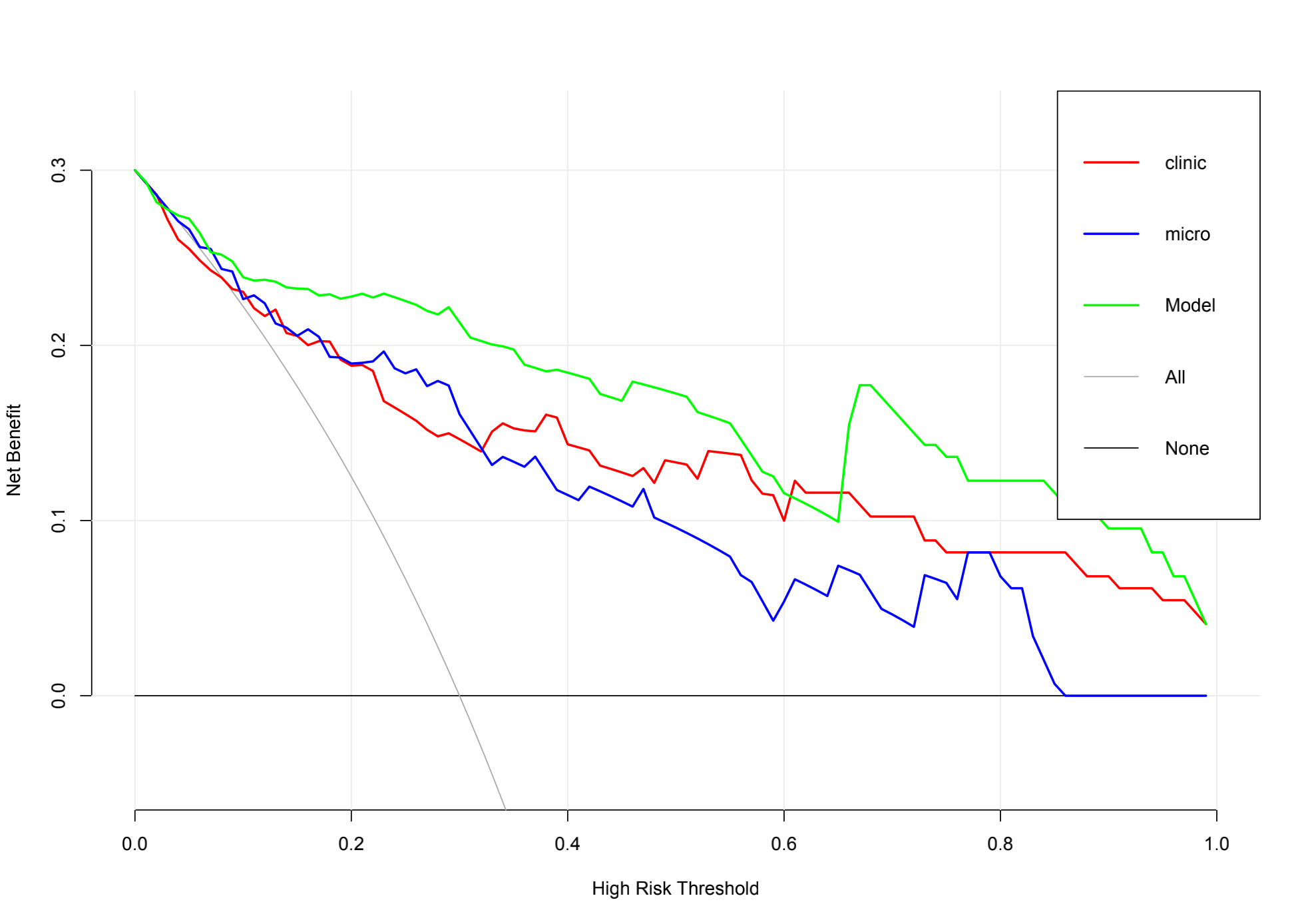


Supplemental figure 2. Decision Curve Analysis of the clinical model ,microbiome model and the combined model.
